# Cognitive recovery in patients with post-stroke subjective cognitive complaints

**DOI:** 10.3389/fneur.2022.977641

**Published:** 2022-09-27

**Authors:** Shaozhen Ji, Hong Sun, Xianglan Jin, Baoxin Chen, Jing Zhou, Jiayi Zhao, Xiao Liang, Wei Shen, Yunling Zhang, Piu Chan

**Affiliations:** ^1^Department of Neurology, Dongfang Hospital, Beijing University of Chinese Medicine, Beijing, China; ^2^Department of Neurobiology, Neurology and Geriatrics, Xuanwu Hospital of Capital Medical University, Beijing Institute of Geriatrics, Beijing, China; ^3^Department of Neurology, Wangjing Hospital, China Academy of Chinese Medical Sciences, Beijing, China; ^4^Department of Neurology, China Academy of Chinese Medical Sciences Xiyuan Hospital, Beijing, China

**Keywords:** cognitive recovery, stroke, post-stroke subjective cognitive complaints, objective cognitive trajectory, post-stroke cognitive impairment

## Abstract

**Background and purpose:**

The objective cognitive trajectory in patients with post-stroke subjective cognitive complaints (SCC) over time remained unknown. We investigated cognitive outcomes in patients with SCC within 1 year after stroke, and determined factors associated with cognitive recovery.

**Methods:**

This study included 599 patients with a clinical diagnosis of post-stroke SCC and evidence of cognitive deficits including Clinical Dementia Rating Scale (CDR) = 0.5, Montreal Cognitive Assessment (MoCA) score <26, and Mini–Mental State Examination score >17 (illiterate) or >20 (primary school) or >24 (junior school or above). Neuropsychological assessment was performed at baseline (2 weeks to 6 months after stroke) and 6-month follow-up visit. Cognitive recovery was operationalized as unimpaired cognition (MoCA score ≥26 and CDR = 0) after 6 months. Factors associated with recovery were defined through logistic regression analysis.

**Results:**

After 6 months, 583 patients completed the follow-up with 80 (13.72%) presenting cognitive recovery, among which, 22 (9.48%) cases reported SCC within 2 weeks after stroke, six (10%) at 15–30 days, 13 (15.12%) at 31–60 days, 10 (16.13%) at 61–90 days, five (10.42%) at 91–120 days, nine (23.08%) at 121–150 days, and 15 (26.79%) at 151–180 days. Compared to those reported cognitive complaints at 151–180 days after stroke, patients with early post-stroke SCC had poorer cognitive recovery, which was only significant in individuals with high level of education. Male sex, higher baseline MoCA scores, coffee intake and thalamus lesions were independently associated with high chance of cognitive recovery.

**Conclusions:**

Although post-stroke SCC contributes to persisting objective cognitive deficits, some patients presented cognitive recovery within 1 year after stroke. Patients with a high education level reporting SCC at earlier stage after stroke had poorer cognitive recovery. Male, higher baseline MoCA scores, coffee intake and thalamus lesions appear to independently predict cognitive recovery.

## Introduction

Subjective cognitive complaints (SCC) are common after stroke with estimated prevalence varying from 28.6 to 92%, defined as whether survivors themselves report cognitive difficulties or problems and if so what these are and whether they are irritating and/or worrying for them (1, 2). Given that the presence of post-stroke SCC was observed significantly correlating with poor cognitive performances (3, 4), it contributes to recognition and diagnosis of post-stroke cognitive impairment (PSCI). However, evidences have shown that post-stroke SCC is inconsistently associated with objective cognitive performance (1, 5), which may be attributed to screening or assessment time of cognitive complaints, since many patients with cognitive disorders after stroke will show recovery over time (4, 6–9). Thus far, most of the studies depend on cross-sectional designs and as such provide limited information about objective cognitive trajectory in patients with post-stroke SCC over time (10). Whether patients with post-stroke SCC will show improvement in cognitive function is still unknown. Identifying patients with potential for cognitive recovery after stroke and predictors may enrich our understanding of the etiology of PSCI and guide diagnosis and individualized rehabilitation intervention.

In the present longitudinal study, we investigated cognitive outcomes in patients with evidence of objective cognitive disorders who reported SCC in the early phase (within 6 months) after stroke, and identified factors related to cognitive recovery. Taking into account the association between SCC and cognitive reserve (CR) (11, 12), we also identified the influence of education on cognitive recovery in patients with post-stroke SCC. Our results are based on the data for the “prospective multi-center cohort study of PSCI” (13).

## Methods and materials

### Study design and procedure

This was a secondary analysis of data for the “prospective multi-center cohort study of PSCI,” which was designed to establish an integrated health management network based on home-based free-living intellectual and physical activities in China for the purpose of improving health and welfare of stroke survivors. In brief, 3,000 patients had acute stroke onset within the prior 6 months were recruited between December 2011 and December 2013 from 14 hospitals of northern and southern China, who had neither pre-stroke cognitive impairment, nor diagnosis of depression or Hamilton Depression Rating Scale (HAMD) score ≥17, nor severe aphasia or serious physical or mental illnesses that could affect neuropsychological assessments. All patients were included according to a clinical diagnosis of stroke, established by at least one experienced neurologist, based on data collected in routine clinical care and imaging. The baseline survey was administered during 14 days to 6 months after acute stroke onset, and the follow-up visit was carried out after 6 months with the same evaluation that comprised a structured clinical work-up and a standardized clinical history and assessments. Face-to-face interviews were conducted by trained physicians and nurses. Informed consent was obtained from participants, and the ethics approval was provided by the research ethics committee of Dongfang Hospital before study initiation (approval number: 2011123004). The study conformed with World Medical Association Declaration of Helsinki.

### Post-stroke SCC

The presence of post-stroke SCC was assessed using self-reports and the Checklist for Cognitive and Emotional consequences following stroke (CLCE-24) inventory, which is a standardized interview exploring post-stroke cognitive, emotional, and behavioral complaints (3). The CLCE-24 consists of 13 cognitive, nine emotional and two non-specified items, which was scored on presence and interference in daily life: zero (SCC not present), one (doubtful presence), two (present, but not affecting daily life), three (present and negatively affecting daily life). Post-stroke SCC were defined as cognitive failures or problems worried and reported by patients themselves after the stroke incident, and at least one “present” or “doubtful presence” item as assessed by the CLCE-24. The time points of the self-perception of SCC were set as 0–14, 15–30, 31–60, 61–90, 91–120, 121–150, and 151–180 days after stroke onset, which was collected through retrospective review to participants conducted at the baseline survey.

### Participants

The current study was based on data obtained at baseline and 6 months follow-up from the “prospective multi-center cohort study of PSCI.” The participants with post-stroke SCC were included in the present study, who were admitted with evidence of cognitive disorder, indicated by the Clinical Dementia Rating Scale (CDR) =0.5, the Montreal Cognitive Assessment (MoCA) score <26, and the Mini–Mental State Examination (MMSE) score >17 (illiterate) or > 20 (primary school) or >24 (junior school or above). The exclusion criteria were as follows: indication of pre-stroke SCC; severe and major depression, or HAMD score ≥17; severe anxiety, aphasia, apraxia, agnosia or other factors that might preclude completion of neuropsychological assessments; and other disorders or use of medication that might affect cognitive functions.

### Data collection and neuropsychological assessment

Demographic characteristics, stroke characteristics, comorbidities and risk factors, cognitive and emotion were surveyed with structural questionnaires by trained physicians and nurses at baseline. Demographic characteristics included age, sex, years of education and body mass index (BMI). Stroke characteristics included stroke severity, location and laterality of stroke lesions, and stroke subtype. Stroke severity was assessed using the National Institutes of Health Stroke Scale (NIHSS). Stroke characteristics including location and Laterality were defined based on CT or MRI. Comorbidities and risk factors included previous diagnosis of hypertension, diabetes, heart disease, hyperlipidemias, and transient ischemic attack (TIA) or stroke, habits of smoking and alcohol drinking. Others including tea and coffee consumption, habits of mobile phone use and physical exercise were also surveyed at baseline. The consumption of smoking, alcohol, tea and coffee consumption was defined as ever (current or former) intake. For mobile phone use and physical exercise, information was collected on frequency (at least 30 min per day) based on a four-point scale (almost every day, 3–4 days per week, 1–2 days per week, and rarely).

Cognitive functioning and emotion status were recorded at the baseline, which included cognition measured with the MoCA, MMSE and CDR, and status of depression after stroke assessed with the HAMD. The ability of daily living was estimated with Activities of Daily Living (ADL) scales.

### Primary outcome assessment

Cognitive recovery was defined as a transition from cognitive disorders to unimpaired cognitive performance after 6 months based on MoCA score ≥26 and CDR=0, which was assessed by at least one experienced neurologist.

### Statistical analysis

Continuous variables were presented as mean ± standard deviation (SD) and compared by Student's *t*-tests, or Kruskal-Wallis tests based on distributional properties. Categorical variables were described as percentages and compared by Chi-square tests. Odds ratio (OR) as the estimates of relative risk for cognitive recovery were calculated using 95% confidence intervals (CI). Univariate and multivariable logistic regression analysis was performed to defined factors associated with cognitive recovery. The full model consisting of the strongest predictors of cognitive recovery, which were firstly determined in univariate analyses (*P* < 0.05). The extended model controlled for confounders related to cognitive recovery selected based on previous literature (7, 14–16). Post-stroke SCC was fit into ordinal logistic regression models as continuous variable by the self-reported time points to calculate *P* for trend.

Interactions between post-stroke SCC and covariates were tested by including cross-product terms in logistic regression models. To investigate whether the association of post-stroke SCC with cognitive recovery differed by education, the study population was divided into low level of education group (years of education <12 years) and high level of education group (years of education ≥12 years). The interaction between education groups and post-stroke SCC (educational level × post-stroke SCC group) with regards to the correlation of cognitive recovery was investigated using univariate and multivariable logistic regression models. Logistic regression models were separately developed to assess the associations between post-stroke SCC and cognitive recovery in both education subgroups (low education group and high education group).

All statistical tests were two-sided, and a *P* value <0.05 was considered statistically significant. Data were analyzed with SPSS version 25.0 (IBM Corp, Armonk, NY, USA).

## Results

A total of 599 patients with post-stroke SCC have been included at baseline. At 2 weeks after stroke onset 40.07% of individuals complained of cognitive difficulties, at 15–30 days 10.18%, at 31–60 days 14.86%, at 61–90 days 10.68%, at 91–120 days 8.01%, at 121–150 days 6.84%, and at 151–180 days 9.35%. Basic demographic and clinical characteristics are shown in Table 1. After 6 months, 16 individuals withdrew or were lost to follow-up (Supplementary Table 1). Finally, 583 (about 97.33%) samples were included in the final analyses (Supplementary Figure 1).

**Table 1:** Characteristics of patients with post-stroke SCC at baseline.

**Characteristics**	***n* = 599**
Demographic characteristics	
Age, mean (SD), y	65.81 ± 10.26
Sex, male, *n* (%)	308 (51.42)
Level of education, *n* (%)	
Low (years of education <12)	346 (57.76)
High (years of education ≥12)	253 (42.24)
BMI, mean (SD)	24.46 ± 3.17
Stroke characteristics	
NIHSS, mean (SD)	1.73 ± 2.48
Location, *n* (%)	
Cerebral cortex	58 (9.68)
Subcortex	154 (25.71)
Basal ganglia	399 (66.61)
Thalamus	39 (6.51)
Brain stem	54 (9.02)
Cerebellum	19 (3.17)
Laterality, *n* (%)	
Left hemisphere	133 (22.20)
Right hemisphere	121 (20.20)
Bilateral hemispheres	309 (51.59)
Lacunar infarction, *n* (%)	344 (57.43)
Hemorrhagic stroke, *n* (%)	28 (4.67)
Comorbidities and risk factors	
Hypertension, *n* (%)	421 (70.28)
Diabetes, *n* (%)	143 (23.87)
Heart disease, *n* (%)	106 (17.70)
Hyperlipidemias, *n* (%)	146 (24.37)
Previous TIA/stroke, *n* (%)	111 (18.53)
Smoking, *n* (%)	198 (33.06)
Alcohol intake, *n* (%)	183 (30.55)
Cognition, emotion and ability of daily living	
MoCA score at baseline, mean (SD)	21.85 ± 3.28
ADL score at baseline, mean (SD)	26.05 ± 10.85
HAMD score at baseline, mean (SD)	3.91 ± 3.68
Other	
Tea intake, *n* (%)	217 (36.23)
Coffee intake, *n* (%)	19 (3.17)
Physical exercise, *n* (%)	
No	104 (17.36)
<1 h per day	400 (66.78)
≥ 1 h per day	95 (15.86)
Mobile-phone use, *n* (%)	435 (72.62)
Reported time of SCC, *n* (%)	
within 14 d	240 (40.07)
15–30 d	61 (10.18)
31–60 d	89 (14.86)
61–90 d	64 (10.68)
91–120 d	48 (8.01)
121–150 d	41 (6.84)
151–180 d	56 (9.35)

SCC, Subjective Cognitive Complaints; BMI, Body Mass Index; TIA, Transient Ischemic Attack; ADL, Activities of Daily Living Scale; HAMD, Hamilton Depression Scale; National Institute of Health Stroke Scale; MMSE, Mini–Mental State Examination; MoCA, Montreal Cognitive Assessment.

**Figure 1:**
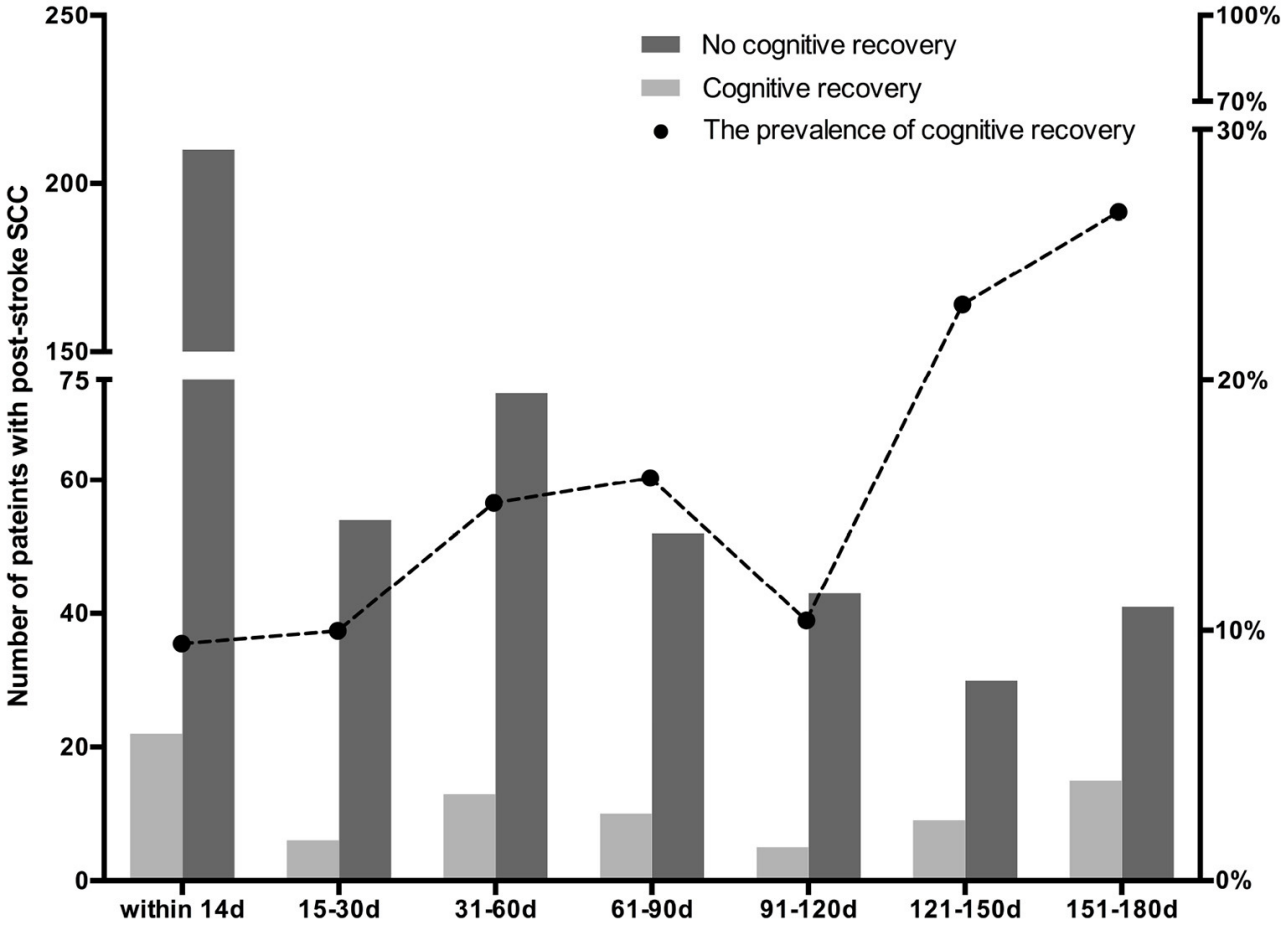
The prevalence of cognitive recovery in patients with subjective cognitive complaints (SCC) at various time periods after stroke.

Eighty (13.72%) Showed Cognitive Recovery After 6 Months, Among Which, 22 (9.48%) Cases Reported to Have Perceived Cognitive Difficulties Within 2 Weeks After Stroke, 6 (10%) at 15–30 Days, 13 (15.12%) at 31–60 Days, 10 (16.13%) at 61–90 Days, 5 (10.42%) at 91–120 Days, 9 (23.08%) at 121–150 Days, and 15 (26.79%) at 151–180 Days (Table 2; Figure 1). There Was a Time-Dependent Trend on Correlation Between Post-Stroke SCC and Cognitive Recovery in Univariate (per Point OR, 0.825 [0.74–0.92]) and Multivariable Models (per Point OR, 0.818 [0.726–0.922]). Compared to Those Reported Cognitive Complaints at 151–180 Days After Stroke, Patients With Post-Stroke SCC at 14 Days, 15–30 Days, and 91–120 Days After Stroke Had Poorer Cognitive Recovery (Table 3).

**Table 2:** Characteristics of patients with cognitive recovery vs. cognitive impairment.

	**Cognitive recovery (*n* = 80)**	**No cognitive recovery (*n* = 503)**	***P-*value**
Demographic characteristics			
Age, mean (SD), y	64.33 ± 10.42	65.97 ± 10.23	0.217
Sex, male, *n* (%)	56 (70.00)	243 (48.31)	<0.001
Level of education, *n* (%)			0.001
Low (years of education <12)	32 (40.00)	301 (59.84)	
High (years of education ≥12)	48 (60.00)	202 (40.16)	
BMI, mean (SD)	24.26 ± 2.62	24.48 ± 3.26	0.736
Stroke characteristics			
NIHSS, mean (SD)	1.65 ± 2.19	1.75 ± 2.53	0.922
Location, *n* (%)			
Cerebral cortex	3 (3.75)	55 (10.93)	0.046
Subcortex	20 (25.00)	130 (25.84)	0.872
Basal ganglia	55 (68.75)	333 (66.20)	0.654
Thalamus	10 (12.50)	29 (5.77)	0.025
Brain stem	2 (2.50)	48 (9.54)	0.037
Cerebellum	4 (5.00)	14 (2.78)	0.287
Laterality, *n* (%)			0.346
Left hemisphere	22 (27.50)	109 (21.67)	
Right hemisphere	17 (21.25)	103 (20.48)	
Bilateral hemispheres	34 (42.5)	262 (52.09)	
Lacunar infarction, *n* (%)	45 (56.25)	287 (57.06)	0.892
Hemorrhagic stroke, *n* (%)	3 (3.75)	24 (4.77)	0.686
Comorbidities and risk factors			
Hypertension, *n* (%)	61 (76.25)	349 (69.38)	0.212
Diabetes, *n* (%)	21 (26.25)	121 (24.06)	0.671
Heart disease, *n* (%)	15 (18.75)	85 (16.90)	0.683
Hyperlipidemias, *n* (%)	21 (26.25)	122 (24.25)	0.7
Previous TIA/stroke, *n* (%)	14 (17.50)	91 (18.09)	0.898
Smoking, *n* (%)	27 (33.75)	165 (32.80)	0.867
Alcohol intake, *n* (%)	26 (32.5)	153 (30.42)	0.708
Cognition, emotion and ability of daily living			
MoCA score at baseline, mean (SD)	23.73 ± 2.20	21.61 ± 3.27	<0.001
ADL score at baseline, mean (SD)	24.29 ± 9.13	26.28 ± 11.05	0.203
HAMD score at baseline, mean (SD)	3.96 ± 3.87	3.91 ± 3.68	0.993
Other			
Tea intake, *n* (%)	31(38.75)	179 (35.59)	0.016
Coffee intake, *n* (%)	6 (7.50)	13 (2.58)	0.021
Physical exercise, *n* (%)			0.837
No	12 (15.00)	87 (17.30)	
<1 h per day	54 (67.50)	337 (67.00)	
≥1 h per day	14 (17.50)	79 (15.70)	
Mobile-phone use, *n* (%)	63 (78.75)	361 (71.77)	0.193
Reported time of SCC, *n* (%)			0.013
within 14 d	22 (9.48)	210 (90.52)	
15–30 d	6 (10)	54 (90)	
31–60 d	13 (15.12)	73 (84.88)	
61–90 d	10 (16.13)	52 (83.87)	
91–120 d	5 (10.42)	43 (89.58)	
121–150 d	9 (23.08)	30 (76.92)	
151–180 d	15 (26.79)	41 (73.21)	

SCC, Subjective Cognitive Complaints; BMI, Body Mass Index; TIA, Transient Ischemic Attack; ADL, Activities of Daily Living Scale; HAMD, Hamilton Depression Scale; National Institute of Health Stroke Scale; MMSE, Mini–Mental State Examination; MoCA, Montreal Cognitive Assessment.

**Table 3:** Factors associated with cognitive recovery in patients with post-stroke SCC.

	**Univariate model**		**Full model** ^**a**^		**Extended model** ^**b**^	
	**OR (95% CI)**	***P*-value**	**OR (95% CI)**	***P*-value**	**OR (95% CI)**	***P*-value**
Reported time of SCC						
151–180 d	Reference group	0.019	Reference group	0.026	Reference group	0.039
121–150 d	0.82 (0.317–2.123)	0.683	0.596 (0.207–1.719)	0.338	0.551 (0.171–1.776)	0.318
91–120 d	0.318 (0.106–0.954)	0.041	0.279 (0.088–0.888)	0.031	0.203 (0.058–0.709)	0.012
61–90 d	0.526 (0.214–1.291)	0.161	0.386 (0.146–1.022)	0.055	0.34 (0.116–0.993)	0.048
31–60 d	0.487 (0.211–1.122)	0.091	0.389 (0.156–0.967)	0.042	0.417 (0.157–1.113)	0.081
15–30 d	0.304 (0.108–0.851)	0.023	0.218 (0.071–0.670)	0.008	0.222 (0.064–0.766)	0.017
within 14 d	0.286 (0.137–0.598)	0.001	0.254 (0.114–0.566)	0.001	0.247 (0.103–0.592)	0.002
***For trend***	0.825 (0.740–0.920)	0.001	0.818 (0.726–0.922)	0.001	0.825 (0.723–0.941)	0.004
Male sex	2.497 (1.500–4.154)	<0.001	2.451 (1.418–4.237)	0.001	4.84 (2.394–9.784)	<0.001
Level of education						
Low (years of education <12)	Reference group		Reference group		Reference group	
High (years of education ≥12)	2.235 (1.381–3.681)	0.001	1.278 (0.741–2.206)	0.378	1.158 (0.623–2.152)	0.644
Location					
Cerebral cortex	0.317 (0.097–1.040)	0.058			0.256 (0.067–0.982)	0.047
Thalamus	2.335 (1.091–4.999)	0.029	2.658 (1.125–6.281)	0.026	2.97 (1.171–7.531)	0.022
Brain stem	0.243 (0.058–1.020)	0.053			0.189 (0.034–1.066)	0.059
MoCA score at baseline	1.478 (1.276–1.712)	<0.001	1.473 (1.258–1.725)	<0.001	1.543 (1.297–1.837)	<0.001
Tea intake	1.145 (0.705–1.861)	0.584			1.123 (0.607–2.075)	0.712
Coffee intake	3.056 (1.127–8.289)	0.028	4.071 (1.271–13.032)	0.018	8.667 (2.378–31.595)	0.001

^*a*^Full Model: adjusted for level of education, sex, coffee intake, lesions location (Cerebral cortex, thalamus, brain stem).

^*b*^Extended Model: adjusted for the variables in (Full model) plus all other comorbidities listed in Table 2.

SCC, Subjective cognitive complaints; MoCA, Montreal Cognitive Assessment; CI, confidence interval; OR, odds ratio.

In univariate analyses, another eight possible factors associated with cognitive recovery in patients with post-stroke SCC were educational level, the MoCA score at baseline, sex, lesions locations (cerebral cortex, thalamus, and brain stem), coffee and tea intake status (Table 2). Of those, five variables (sex, educational level, the MoCA score at baseline, thalamus lesions, coffee intake) met the threshold of *P* < 0.05 in the preselection of conventional factors for univariate logistic regression models. The selection of these five factors and reported time of post-stroke SCC in multivariable analysis resulted in the full model with five variables as the strongest factors related to cognitive recovery: sex (men compared with women: OR, 2.451 [1.418–4.237]), the MoCA score at baseline (per point OR, 1.473 [1.258–1.725]), coffee intake (coffee consumption compared with no-coffee consumption: OR, 4.071 [1.271–13.032]), and thalamus stroke (thalamus lesions compared with no- thalamus lesions: OR, 2.658 [1.125–6.281]). And the associations were remained significant after controlling for multiple confounders in the extended model (Table 3).

An interaction was identified between educational level and post-stroke SCC in univariate and multivariable models. After stratifying the study population into two subgroups according to the level of education, we found that the time-dependent trend on association between post-stroke SCC and cognitive recovery was only significant in the individuals with high level of education (per point OR, 0.793 [0.679–0.925]) (Table 4).

**Table 4:** Association between SCC reported in various time periods after stroke and cognitive recovery according to education level.

**Reported time of post-stroke SCC**	**151–180 d**	**121–150 d**	**91–120 d**	**61–90 d**	**31–60 d**	**15–30 d**	**within 14 d**	***For tend***
Patients with high level of education (*n* = 250)							
Cognitive recovery, *n* (%)	8 (16.67)	8 (16.67)	2 (4.17)	9 (18.75)	7 (14.58)	5 (10.42)	9 (18.75)	/
No cognitive recovery, *n* (%)	14 (6.93)	13 (6.44)	23 (11.39)	24 (11.88)	31 (15.35)	25 (12.38)	72 (35.64)	/
OR	Reference group	1.077	0.152	0.656	0.395	0.35	0.219	0.793
95% CI	Reference group	0.312–3.71	0.028–0.821	0.206–2.09	0.12–1.305	0.096–1.278	0.072–0.665	0.679–0.925
*P-*value	0.024	0.907	0.029	0.476	0.128	0.112	0.007	0.003
Patients with low level of education (*n* = 333)							
Cognitive recovery, *n* (%)	7 (21.88)	1 (3.13)	3 (9.38)	1 (3.13)	6 (18.75)	1 (3.13)	13 (40.63)	/
No cognitive recovery, *n* (%)	27 (8.97)	17 (5.65)	20 (6.64)	28 (9.3)	42 (13.95)	29 (9.63)	138 (45.85)	/
OR	Reference group	0.227	0.579	0.138	0.551	0.133	0.363	0.879
95% CI	Reference group	0.026–2.01	0.133–2.519	0.016–1.196	0.167–1.816	0.015–1.153	0.133–0.995	0.746–1.036
*P-*value	0.262	0.183	0.466	0.072	0.327	0.067	0.049	0.123

SCC, Subjective cognitive complaints; CI, confidence interval; OR, odds ratio.

## Discussion

Although post-stroke SCC is generally studied in the context of progressing to objective cognitive impairment, not all survivors with SCC at an early phase following stroke would exhibit constant cognitive dysfunction. Our results showed that the improvement of objective cognitive performance after stroke did indeed take place in 14% of patients with post-stroke SCC, which is in agreement with some previous studies showing that 10–15% of the stroke patients had recovered from cognitive impairment no dementia after 1 year (17, 18). Considering post-stroke SCC is associated with psychological factors such as depression, anxiety, perceived stress and coping style (5, 19–21), our findings suggested that psychological distress due to stroke incident might play an important role on acute deficiencies in cognitive test scores or immediate cognitive syndromes in patients with post-stroke SCC.

In addition, we found that chances of cognitive recovery varied among patients with SCC reported in various time periods after stroke, and patients with SCC at an earlier stage after stroke might have poorer cognitive recovery, which suggested that those individuals might have more irreversible cognitive impairments or negatively potential for cognitive improvement. More interestingly, this association between post-stroke SCC and cognitive recovery was only observed in individuals with high education. The present study showed SCC at an earlier stage after stroke might be indicative of irreversibly impaired cognitive performance in patients with high education. Education is defined as one proxy for CR, which reflects the brain resilience in response to neuropathological changes (22). This result suggested that high CR could contribute to early and sensitively self-perception on irreversible cognitive impairment following stroke, which might be a message to ask for early intervention for improvement in cognitive function. In line with the previous researches among elders with subjective cognitive decline (23) and stroke survivors (24), high CR might be a protective factor against progression to dementia and for promoting cognitive recovery in patients with post-stroke SCC.

Evidence is emerging on prediction of cognitive recovery after stroke (14, 25), however, information about factors associated with cognitive recovery in patients with post-stroke SCC is limited. This study indicated cognitive recovery in patients with post-stroke SCC was independently associated with sex, baseline MoCA score, coffee intake, and thalamus lesions. Contrary to some earlier researches (17, 26), we observed male patients with post-stroke SCC got a greater chance of cognitive recovery. Potential pathways might include psychological, social, and cultural factors that shape attitudes, behaviors, and knowledge (27), in which men have more advantages than women. We found that patients who reported SCC following stroke but had higher MoCA scores at baseline got a greater chance of cognitive recovery. Although some studies showed that the evolution of post-stroke SCC was associated with psychological resilience (21), our results indicated the influence of baseline cognitive performance on improvement in objective cognitive function. Given that we only evaluated global cognitive function without cognitive performance in domains, it remained unclear whether impaired cognitive domains related to characteristics of vascular cognitive impairment correlated with cognitive recovery in patients with post-stroke SCC, such as visuospatial/executive function, attention, orientation and language (25). Coffee is the primary source of caffeine and contains phenolics and other bioactive compounds with potential benefit for several neurological diseases (28). Few studies have investigated the relationship between coffee intake and vascular dementia with inconsistent observations, possibly due to the sample size (29, 30). Our findings supported that coffee consumption seemed to correlate with high chance of cognitive recovery in patients with post-stroke SCC. Further exploration is required to identify component responsible for the beneficial effect of coffee, such as caffeine and phenolic acids, to develop medication for improving cognitive abilities after stroke. Ample literature has consistently showed acute and long-term cognitive disorders (31–34) despite initial improvements (35) in patients with thalamic stroke, which is possibly attributed to impairments of frontal lobe-related functions. In this study, we found thalamus infarctions were associated with cognitive recovery in the early phase after stroke among patients with post-stroke SCC. These patients with thalamic stroke presented with early cognitive improvements, perhaps because infarction areas of almost all patients were not in the anterior area (33, 34), and seven patients had right-sided lesions in 10 subjects with objective cognitive recovery (32, 35). However, there was no appropriate explanation for this observation due to lack of long-term investigation and the detail of neuroimaging data.

The current study is one of few longitudinal studies to investigate objective cognitive performances during 12 months after stroke in patients with post-stroke SCC. A strength of our study is that we included patients with post-stroke SCC within 6 months after stroke. Better prognostication of cognitive recovery in this phase is relevant for daily practice. Moreover, nearly all patients in this study completed six-month follow-up, which limits the effects of bias from loss to follow-up. However, the current study has some limitations. First, despite the substantial cohort size for PSCI, sample size was still modest for the purpose of demonstrating factors associated with cognitive recovery in patients with post-stroke SCC. Second, the results were based on the data of participants without severe depression and anxiety, in light of the interaction between post-stroke SCC and mood (depression and anxiety) (20, 36), but we did not investigate the association between the severity of post-stroke SCC, two components of SCC (SCC-content vs. SCC-worry) and cognitive recovery. Furthermore, although a variety of covariates was adjusted, residual confounding by measurement error or unmeasured factors (such as recurrent stroke, lesion size, treatment factors including rehabilitation exercise) could not be avoided.

## Conclusion

Although post-stroke SCC correlates with persisting objective cognitive impairment, there is still a few patients with SCC at an early phase following stroke presented cognitive recovery. Patients with a high education level who reported SCC at an earlier stage after stroke had poorer cognitive recovery. And some factors appear to independently predict cognitive recovery in patients with post-stroke SCC, including male sex, high baseline MoCA score, coffee consumption, and thalamus stroke, which may contribute to intervention.

## Data availability statement

The raw data supporting the conclusions of this article will be made available by the authors, without undue reservation.

## Ethics statement

The studies involving human participants were reviewed and approved by the Ethical Committee of Dongfang hospital, Beijing University of Chinese Medicine (approval number: 2011123004). The patients/participants provided their written informed consent to participate in this study.

## Author contributions

YZ had full access to all of the data in the study and takes responsibility for the integrity of the data and the accuracy of the data analysis. SJ, PC, and YZ contributed to the study concept and design. SJ, XJ, BC, JiaZ, and WS contributed to acquisition, analysis, or interpretation of data. SJ contributed to the drafting of the manuscript and statistical analysis. PC and YZ contributed to the critical revision of the manuscript for important intellectual content and study supervision. SJ, HS, BC, and YZ obtained funding. HS, BC, JinZ, XL, and YZ contributed to the administrative, technical, or material support. All authors contributed to the article and approved the submitted version.

## Funding

This study was supported by National TCM Leading Personnel Support Program [NATCM Personnel and Education Department (2018)] (No. 12), the Innovation Team and Talents Cultivation Program of National Administration of Traditional Chinese Medicine (No. ZYYCXTD-C-202007), National Key R&D Program of China (2018YFC1704303), Prevention and Intervention Projects Program of Stroke and Post-stroke Disability in Elderly by National Health Commission of People's Republic of China, and the Fundamental Research Funds for the Central Universities of China (2021-JYB-XJSJJ071).

## Conflict of interest

The authors declare that the research was conducted in the absence of any commercial or financial relationships that could be construed as a potential conflict of interest.

## Publisher's note

All claims expressed in this article are solely those of the authors and do not necessarily represent those of their affiliated organizations, or those of the publisher, the editors and the reviewers. Any product that may be evaluated in this article, or claim that may be made by its manufacturer, is not guaranteed or endorsed by the publisher.
