# Supplementary material for: Cognitive recovery in patients with post-stroke subjective cognitive complaints
Source: Front Neurol. 2022 Sep 27;13:977641. doi: 10.3389/fneur.2022.977641 (PMC9551021; doi:10.3389/fneur.2022.977641)
Supplement: Supplementary file 1 [file Table_1.docx]

Supplementary Table 1 Characteristics of subjects included vs not included in final analyses

|  | Final sample(n=583) | Lost to follow-up (n=16) | *P* value |
| --- | --- | --- | --- |
| Demographic characteristics |  |  |  |
| Age, mean (SD), y | 65.74 ± 10.27 | 68.13 ± 10.04 | 0.33 |
| Sex, male, n (%) | 299 (51.29) | 9 (56.25) | 0.695 |
| Level of education, n (%) |  |  | 0.054 |
| Low (years of education <12) | 13 (3.76) | 333 (96.24) |  |
| High (years of education ≥12) | 3 (1.19) | 250 (98.81) |  |
| BMI, mean (SD) | 65.74 ± 10.27 | 68.13 ± 10.04 | 0.33 |
| Stroke characteristics |  |  |  |
| NIHSS, mean (SD) | 1.74 ± 2.49 | 1.31 ± 2.39 | 0.476 |
| Location, n (%) |  |  |  |
| Cerebral cortex | 58 (9.95) | 0 (0) | 0.184 |
| Subcortex | 150 (25.73) | 4 (25) | 0.984 |
| Basal ganglia | 388 (66.55) | 11 (68.75) | 0.854 |
| Thalamus | 39 (6.69) | 0 (0) | 0.285 |
| Brain stem | 50 (8.58) | 4 (25) | 0.024 |
| Cerebellum | 18 (3.09) | 1 (6.25) | 0.476 |
| Laterality, n (%) |  |  | 0.108 |
| Left hemisphere | 131 (22.47) | 2 (12.5) |  |
| Right hemisphere | 120 (20.58) | 1 (6.25) |  |
| Bilateral hemispheres | 296 (50.77) | 13 (81.25) |  |
| Lacunar infarction, n (%) | 332 (56.95) | 12 (75) | 0.15 |
| Hemorrhagic stroke, n (%) | 27 (4.63) | 1 (6.25) | 0.762 |
| Comorbidities and risk factors |  |  |  |
| Hypertension, n (%) | 410 (70.33) | 11 (68.75) | 0.892 |
| Diabetes, n (%) | 142 (24.36) | 1 (6.25) | 0.094 |
| Heart disease, n (%) | 100 (17.15) | 6 (37.5) | 0.035 |
| Hyperlipidemias, n (%) | 143 (24.53) | 3 (18.75) | 0.595 |
| Previous TIA/stroke, n (%) | 105 (18.01) | 6 (37.5) | 0.048 |
| Smoking, n (%) | 192 (32.93) | 6 (37.5) | 0.702 |
| Alcohol intake, n (%) | 179 (30.70) | 4 (25) | 0.625 |
| Cognition, emotion and ability of daily living | | | |
| MoCA score at baseline, mean (SD) | 21.90 ± 3.22 | 20.06 ± 4.58 | 0.111 |
| ADL score at baseline, mean (SD) | 26.01 ± 10.82 | 27.44 ± 12.18 | 0.917 |
| HAMD score at baseline, mean (SD) | 3.92 ± 3.70 | 3.63 ± 2.90 | 0.979 |
| Other |  |  |  |
| Tea intake, n (%) | 210 (36.02) | 7 (43.75) | 0.526 |
| Coffee intake, n (%) | 19 (3.29) | 0 (0) | 0.463 |
| Physical exercise, n (%) |  |  | 0.33 |
| No | 99 (16.98) | 5 (31.25) |  |
| < 1 h per day | 391 (67.07) | 9 (56.25) |  |
| ≥ 1 h per day | 93 (15.95) | 2 (12.5) |  |
| Mobile-phone use, n (%) | 424 (72.73) | 11 (68.75) | 0.725 |
| Reported time of SCC, n (%) |  |  | 0.61 |
| within 14 d | 232 (39.79) | 8 (20) |  |
| 15-30 d | 60 (10.29) | 1 (6.25) |  |
| 31-60 d | 86 (14.75) | 3 (18.75) |  |
| 61-90 d | 62 (10.63) | 2 (12.5) |  |
| 91-120 d | 48 (8.23) | 0 (0) |  |
| 121-150 d | 39 (6.69) | 2 (12.5) |  |
| 151-180 d | 56 (9.61) | 0 (0) |  |

SCC: Subjective Cognitive Complaints; BMI: Body Mass Index; TIA: Transient Ischemic Attack; ADL: Activities of Daily Living Scale; HAMD: Hamilton Depression Scale; National Institute of Health Stroke Scale; MMSE: Mini–Mental State Examination; MoCA: Montreal Cognitive Assessment.
