# Supplementary material for: Cognitive recovery in patients with post-stroke subjective cognitive complaints
Source: Front Neurol. 2022 Sep 27;13:977641. doi: 10.3389/fneur.2022.977641 (PMC9551021; doi:10.3389/fneur.2022.977641)
Supplement: Supplementary file 2 [file Image_1.pdf]

Supplementary Figure 1 Flow-chart of participants flow

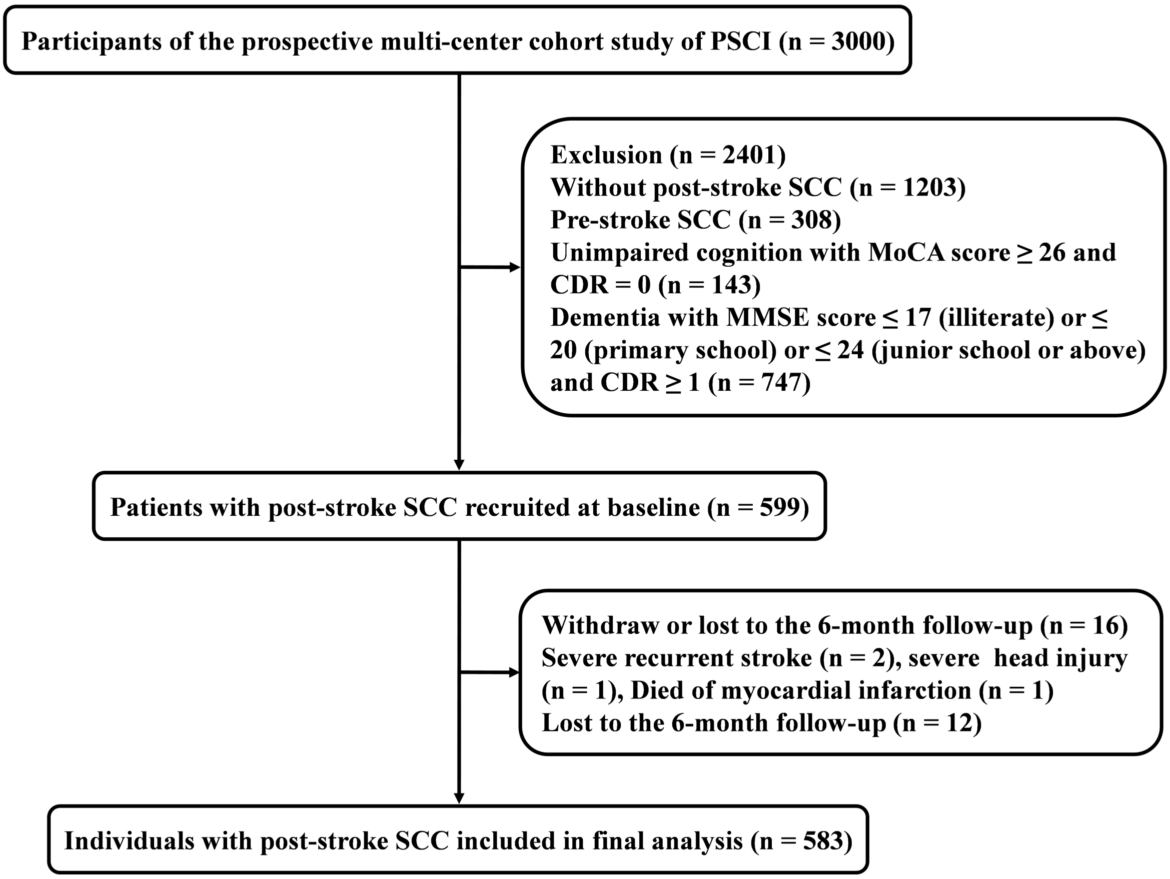

PSCI: Post-stroke cognitive impairment; SCC: Subjective Cognitive Complaints; MoCA: Montreal Cognitive Assessment; MMSE: Mini–Mental State Examination; CDR: Clinical Dementia Rating Scale.
